# *Lactobacillus rhamnosus* GG Affects Microbiota and Suppresses Autophagy in the Intestines of Pigs Challenged with *Salmonella* Infantis

**DOI:** 10.3389/fmicb.2017.02705

**Published:** 2018-01-17

**Authors:** Wei Zhang, Yao-Hong Zhu, Gui-Yan Yang, Xiao Liu, Bing Xia, Xiong Hu, Jin-Hui Su, Jiu-Feng Wang

**Affiliations:** Department of Veterinary Clinical Sciences, College of Veterinary Medicine, China Agricultural University, Beijing, China

**Keywords:** *Lactobacillus rhamnosus*, *Salmonella* Infantis, gut microbiota, autophagy, EGFR/Akt, pig

## Abstract

*Salmonella enterica* serovar Infantis (*S*. Infantis) is a common source of foodborne gastroenteritis worldwide. Here, *Lactobacillus rhamnosus* GG (LGG) was administrated to weaned piglets for 1 week before *S*. Infantis challenge. *S*. Infantis caused decreased ileal mucosal microbiota diversity, a dramatic *Lactobacillus amylovorus* bloom, and decreased abundance of *Arsenicicoccus, Janibacter, Kocuria, Nocardioides, Devosia, Paracoccus, Psychrobacter*, and *Weissella*. The beneficial effect of LGG correlated with the moderate expansion of *L. amylovorus, L. agilis*, and several members of the phyla *Proteobacteria, Firmicutes*, and *Bacteroidetes*. *S*. Infantis translocation to the liver was decreased in the LGG-pretreated piglets. An *in vitro* model of LGG and *S*. Infantis co-incubation (involving the porcine intestinal epithelial cell line IPEC-J2) was established, and nalidixic acid was used to kill the extracellular *S*. Infantis. LGG suppressed the initial *S*. Infantis invasion in the IPEC-J2 cells and deceased the rate of cell death. LGG inhibited *S*. Infantis-induced autophagy and promoted epidermal growth factor receptor (EGFR) and Akt phosphorylation in both the ileum and IPEC-J2 cells. Our findings suggest that LGG inhibited *S*. Infantis-induced autophagy by promoting EGFR-mediated activation of the negative mediator Akt, which, in turn, suppressed intestinal epithelial cell death and thus restricted systemic *S*. Infantis infection. LGG can restore the gut microbiota balance and preserve the autophagy-related intestinal epithelial barrier, thereby controlling infections.

## Introduction

Salmonellosis is the most prevalent bacterial foodborne zoonosis in humans (Tran et al., 2018) and is estimated to cause an overall incidence of 49 cases/100,000 individuals and 3.4 million cases of non-typhoidal *Salmonella* disease each year (Ao et al., 2015). *Salmonella enterica* subsp. *enterica* serovar Infantis (*S*. Infantis) is a highly frequent cause of persistent Salmonellosis compared to other serovars (Marzel et al., 2016). Importantly, *S*. Infantis-contaminated pork products are responsible for acute foodborne gastroenteritis in humans (Schroeder et al., 2016), which makes swine salmonellosis a major public health concern.

The *S*. Infantis strains frequently isolated from weaned piglets have been found to be multidrug resistant, including to sulfamethoxazole and trimethoprim, which are frequently used in the swine industry (Ikwap et al., 2014). Due to the serious public health concern regarding the spread of multidrug-resistant bacteria, finding antibiotic alternatives to maintain piglet health has become an emergency. The probiotic *Lactobacillus rhamnosus* GG (LGG) are a lactic acid bacteria known to be beneficial for human and animal health. Several possible mechanisms underlying the beneficial effects of LGG have been largely investigated, including inhibition of pathogen biofilm formation (Petrova et al., 2016), competitive exclusion of pathogen via mucus-binding pilin (Ardita et al., 2014; Tytgat et al., 2016), as well as modulating the gut microbiome (Canani et al., 2016; Durack et al., 2017), or maintenance of intestinal barrier function (Khailova et al., 2017) and regulation of host immune response (Johansson et al., 2016). Our recent study found that administration of LGG reduces the severity of *S*. Infantis-induced diarrhea and ameliorates intestinal inflammation in newly weaned piglets (Yang et al., 2017). However, the pathogenesis of *S*. Infantis and the exact mode of action of LGG against *S*. Infantis infection remain unknown.

The gut microbiota endows the host with additional functional features, such as pathogen displacement and resistance against pathogen colonization. Restoration of the commensal gut microbiota by intestinal alkaline phosphatase reduces *S*. Typhimurium colonization (Malo et al., 2010). Using the next-generation high-throughput sequencing of the 16S ribosomal RNA (rRNA) gene, we found that probiotic supplementation is a potential microbiota-related strategy for managing enteric infection (Zhang et al., 2017). However, the effect of *L. rhamnosus* on *S*. Infantis-induced reprogramming of the ileal mucosal microbiota in pigs and the potential contribution of *L. rhamnosus* to *S*. Infantis infection remain unclear.

Intestinal epithelial cells (IECs) serve as the first line of defense against bacterial invasion of host tissues. Autophagy is an evolutionarily conserved cellular pathway that involves cytoplasmic materials being sequestered inside double-membraned autophagosomes and delivered to lysosomes for degradation (Huang and Brumell, 2014). A hallmark of autophagosome formation is the conversion of the cytosolic inactive microtubule-associated protein 1 light chain 3 (LC3)-I into the lipidated active LC3-II that associates with autophagosome membranes. Recently, autophagy has also emerged as an IEC-autonomous mechanism against invasive pathogens (Benjamin et al., 2013). In mice, autophagy in the IECs can be induced by *S*. Typhimurium infection, and it promotes bacterial clearance (Conway et al., 2013). However, *S*. Typhimurium can obtain host-derived peptides to allow it to replicate rapidly in IECs (Singh et al., 2017). In contrast to autophagy-induced cell survival, autophagy can also contribute to autophagic IEC death under certain stress conditions due to prolonged autophagy or over-stimulated autophagy (Levine and Kroemer, 2008; Tang et al., 2015). The process by which *S*. Infantis induces autophagy in IECs and the role of autophagy in the onset of infection are still unclear.

In response to growth factors or nutrient status, phosphatidylinositol-4,5-bisphosphate 3-kinase (PI3K)/Akt serves as an important upstream regulator of the mechanistic target of rapamycin (mTOR), which functions as a master regulator of autophagy (Choi et al., 2013). Epidermal growth factor receptor (EGFR) signaling plays an important role in regulating cellular homeostasis. The indirect recruitment of PI3K to tyrosine-phosphorylated EGFR activates the downstream target, Akt. Recent studies have shown a role for EGFR signaling in autophagy inhibition by activation of the PI3K/Akt/mTOR pathway in IECs and cancer cells (Maynard et al., 2010; Wei et al., 2013; Tan et al., 2015). In our previous study using a porcine IPEC-J2 IEC model of *E. coli* infection, *L. rhamnosus* pretreatment suppressed EGFR activation and enhanced Akt activation (Zhang et al., 2015).

In this study, we carried out a phylotype analysis to examine the effect of the probiotic LGG on the ileal mucosal microbiota during *S*. Infantis infection in newly weaned piglets. We also tested the hypothesis that EGFR/Akt signaling is involved in the mechanism by which LGG regulates *S*. Infantis-induced autophagy in IPEC-J2 cells.

## Materials and methods

### Ethics statement

All animals were treated in strict accordance with the *Guidelines for Laboratory Animal Use and Care* from the Chinese Center for Disease Control and Prevention and the *Rules for Medical Laboratory Animals* (1998) from the Chinese Ministry of Health, under protocol CAU20151001-1, which was approved by the Animal Ethics Committee of the China Agricultural University. All surgeries were performed under xylazine hydrochloride anesthesia, and every effort was made to minimize suffering.

### Bacterial strains

LGG ATCC 53103 (Gefilus, Valio Ltd., Helsinki, Finland) was grown in De Man, Rogosa, and Sharpe (MRS) broth (Oxoid, Basingstoke, UK) for 24 h at 37°C under microaerophilic conditions. For all experiments, after overnight incubation, the bacteria were inoculated 1:100 in fresh MRS broth and grown for about 8 h until reaching mid-log phase.

*Salmonella enterica* serovar Infantis strain CAU1508 was isolated from the intestinal contents of weaned pigs with diarrhea in our laboratory, as previously described (Yang et al., 2017). The *S*. Infantis CAU1508 strain was positive for virulence factors *SipA, SipB, SopA, SopB, SopD, SopE, SopE2, FljB*, and *SseI*. An *S*. Infantis strain harboring the pFPV-mCherry plasmid (for constitutive expression of mCherry) was created as previously described (Drecktrah et al., 2008).

### Antibiotic susceptibility testing and minimal inhibitory concentration (MIC) and minimum bactericidal concentration (MBC) determination

*S*. Infantis and LGG antibiotic susceptibility testing were performed on Mueller Hinton (MH) and MRS agar plates according to the Kirby Bauer disc diffusion method. The tested antibiotics included nalidixic acid (NAL), gentamicin, kanamycin, amikacin, tobramycin, ciprofloxacin, amoxicillin, ampicillin/sulbactam, cefazolin, ceftriaxone, cefotaxime, meropenem, nitrofurantoin, aztreonam, chloromycetin, erythromycin, vancomycin, and oxacillin (China Institute of Veterinary Drugs Control, Beijing, China). Based on the susceptibility testing results, standardized broth dilution MIC and MBC assays of NAL were carried out, as previously described (Condell et al., 2012).

### Experimental design

The treatment regimens and challenge procedure for the pigs were performed as described elsewhere (Yang et al., 2017). In brief, on the day of weaning (day 0), the pigs were assigned to three groups (*n* = 7 per group). Each group received a different treatment, as follows: (1) CONT group, oral administration of sterile physiological saline for 7 days; (2) SI group, oral administration of sterile physiological saline for 7 days and oral challenge with mCherry-*S*. Infantis on day 8 (5.0 × 10^10^ CFU/ml, 10 ml); (3) LGG+SI group, oral administration of LGG (1 × 10^9^ CFU/ml, 10 ml/day) for 7 days and oral challenge with mCherry-*S*. Infantis on day 8 (5.0 × 10^10^ CFU/ml, 10 ml). At 0900 a.m., on days 1 to 7, the pigs in the LGG+SI group were intragastrically administrated 10 ml LGG solution daily, while pigs in the CONT and SI groups were pretreated with an equal volume of sterile physiological saline. At 0900 a.m. on day 8, the pigs in groups SI and LGG+SI were orally challenged with 10 ml of mCherry-*S*. Infantis, whereas the pigs in the CONT group received 10 ml of sterile physiological saline. On day 18, the pigs in the three groups were sacrificed.

### 16S rRNA gene sequencing and phylotype analysis

The ileal mucosal samples were obtained by scraping them from the luminal surfaces using a sterile glass microscope slide, as previous described (Looft et al., 2014), and the total bacterial genomic DNA was extracted using a QIAamp DNA Microbiome kit (Qiagen, Hilden, Germany) according to the manufacturer's instructions. Amplification and sequencing of the V3-V4 region of the bacterial 16S rRNA genes and phylotype analysis was performed as previously described (Zhang et al., 2017). In brief, the bacterial genomic DNA was amplified with the 338F (5′-ACT CCT ACG GGA GGC AGC AG-3′) and the 806R (5′-GGA CTA CHV GGG TWT CTA AT-3′). After amplicons cleaned and quantified, equimolar doses were pooled and paired-end sequenced (PE 2 × 300) on an Illumina Miseq platform according to the manufacturer's protocols. Raw fastq data were demultiplexed and quality filtered using the Quantitative Insights Into Microbial Ecology (QIIME) pipeline (http://qiime.org). Chimera sequences were detected and removed from the denoised sequences against the Silva Gold reference database (release 115; http://www.arb-silva.de/) using the Uchime algorithm. The high-quality sequences were clustered into operational taxonomic units (OTUs) at a 97% nucleotide similarity level using Usearch 6.1 methodology (http://qiime.org/). The most abundant sequences from each OUT were assigned to taxonomic classification against the Silva (SSU117/119) database at a bootstrap cutoff of 80% via the RDP classifier version 2.2 (http://sourceforge.net/projects/rdp-classifier). The alpha diversity (Shannon and Simpson), richness (ACE and Chao1), rarefaction curve, and Venn diagram analysis were performed using Mothur version 1.31.2 (http://www.mothur.org). A heatmap was generated on the basis of the relative abundance of OTUs using R version2.15 (http://www.R-project.org). The linear discriminant analysis (LDA) effect size (LEfSe) method was used to identify indicator bacteria that differentiated the treatment-specific microbiota features. Multivariate data analysis, including a Venn analysis and partial least squares-discriminate analysis (PLS-DA) was performed using R (version 3.2.1) and Simca-*P* 12.0 (Umetrics, Umea, Sweden), respectively.

### PCR quantification of *Lactobacillus species*

Multiple alignments of the 16S rRNA gene of different *Lactobacillus* species obtained from GenBank database were constructed by CLUSTAL W method using Megalign program (DNAStar). Specific sequences were identified to design primer sets for the species: *L. agilis* (F: 5′- GCT ATC TGC AGT CGA CGC TTT-3′, R: 5′-GCGGTCATCATGGTTATGCG-3′), *L. faecis* (5′-CCGAAAGAGGGGGATAACAC-3′, R: 5′-CCGAAACCACCTTTCACATAAAC-3′), and *L. amylovorus* (F: 5′-AAGCTGTCGCTAAGGGATGG-3′, R: 5′-ATGCATCGTCGCCTTGGTAA-3′). Specificity of the primer sets was analyzed using the BLAST algorithm.

Quantitative PCR was performed using an ABI7500 real-time PCR system (Applied Biosystems, Foster City, CA), as previously described (Zhang et al., 2017). Each run included a standard curve and each sample was run in triplicate. Bacterial contents were interpolated from a standard curve and then weight corrected to yield a value in 16S rRNA gene copy number/g mucus.

### Determination of *Salmonella* dissemination in internal organs

Using 5-mm glass beads and a tissue homogenizer (Thomas Scientific, Swedesboro, NJ), 1 g of samples of liver and spleen were minced and then homogenized with 9 ml buffered peptone water for 3 min at high speed. Serial dilutions were then plated on Xylose Lactose Tergitol 4 agar plates and incubated for 24 h at 37°C under aerobic conditions. The results are expressed as log_10_ CFU/g liver or spleen. All counts were performed in triplicate.

### IPEC-J2 cell culture

Porcine intestinal epithelial IPEC-J2 cells were cultured as previously described (Zhang et al., 2015). The cells were subjected to four conditions: (i) medium alone; (ii) *S*. Infantis alone (3 × 10^8^ CFU) at a multiplicity of infection (MOI) of 600:1; (iii) incubation with LGG (3 × 10^8^ CFU) for 2 h, or (iv) preincubation with LGG (3 × 10^8^ CFU) for 2 h prior to exposure to *S*. Infantis. At 2 h after preincubation with LGG, the IPEC-J2 cells were washed three times with phosphate-buffered saline (PBS) and immediately infected with *S*. Infantis for 2 h, followed by three rinses with PBS to remove the non-adherent bacteria. Growth medium supplemented with 50 μg/ml of NAL was added for 1 h (to kill any remaining extracellular *S*. Infantis), following by growth medium containing 10 μg/ml of NAL for the remainder of the experiment (to restrict the extracellular growth of *S*. Infantis).

### Enumeration of intracellular *S*. infantis

*S*. Infantis internalization assays were performed as previously described (Wu et al., 2015). Briefly, confluent IPEC-J2 monolayer cells were incubated with (i) Dulbecco's Modified Eagle's Medium (DMEM), (ii) live LGG (3 × 10^8^ CFU), (iii) ultraviolet-irradiated LGG, (iv) heat-killed LGG, (v) LGG supernatant (pH 7.2), or (vi) medium acidified with lactic acid (pH 6.8). At 2 h after pretreatment, the cells were infected with *S*. Infantis (3 × 10^8^ CFU), as described above. After the growth medium containing 50 μ g/ml of NAL was removed, the number of initial intracellular *S*. Infantis recovered was determined on Luria-Bertani agar.

### Immunofluorescence

IPEC-J2 cells seeded on glass coverslips were infected with mCherry-*S*. Infantis (3 × 10^8^ CFU), as described above. After the growth medium containing 50 μg/ml of NAL was removed, the cells were washed, fixed with 4% paraformaldehyde for 15 min on ice, permeabilized with 0.2% (vol/vol) Triton X-100 (Sigma-Aldrich, Saint Louis, MO) and blocked with 1% bovine serum albumin. Subsequently, the cells were incubated with mouse anti-cytokeratin-18 primary monoclonal antibody at a dilution of 1:200 (Ab668; Abcam, Cambridge, UK) for 45 min at 4°C, following by secondary antibody goat anti-mouse fluorescein isothiocyanate-conjugated IgG (F4143; Sigma-Aldrich). The cell nuclei were stained using 4',6'-diamidino-2-phenylindole (DAPI; Sigma-Aldrich). The recovered *S*. Infantis in the liver from SI pigs were determined by staining frozen sections of liver with the rabbit anti-pig α-tubulin primary monoclonal antibody (1:200 dilution, ab52866, Abcam), secondary antibody goat anti-rabbit FITC-conjugated IgG (F6005, Sigma-Aldrich) and DAPI. The coverslips and slides were visualized and photographed under an inverted Nikon Eclipse Ti-U fluorescence microscope equipped with a Nikon DS cooled camera head (Nikon, Tokyo, Japan).

### Scanning and transmission electron microscopy

At 6 h after *S*. Infantis challenge, the IPEC-J2 cells were harvested and fixed with 3% glutaraldehyde (pH 7.4). The cells were observed using scanning and transmission electron microscopy, as previously described (Wu et al., 2015).

### Cell death assay

At 2, 6, and 12 h after *S*. Infantis challenge, the IPEC-J2 cells were harvested and mixed with 0.4% Trypan blue dye (Invitrogen, Carlsbad, CA). Under a light microscope, dead cells are blue in color, and they were counted using a hemocytometer. The results are presented as the ratio of the number of dead cells to the total number of cells.

### Western blotting

Proteins were extracted from the jejunum and ileum samples and IPEC-J2 cells, as previously described (Zhang et al., 2015; Yang et al., 2017). The primary antibodies were rabbit anti-phospho-Ser473 (p)-Akt (1:1000, ab138726), rabbit anti-phospho-Tyr1068 (p)-EGFR (1:250, ab32430; Epitomics, Burlingame, CA), rabbit anti-LC3A/B (1:1000, Cell Signaling Technology, Danvers, MA), rabbit anti-total-EGFR (1:500, 18986-1-AP), rabbit anti-total-Akt (1:1000, 10176-2-AP), and mouse anti-glyceraldehyde-3-phosphate dehydrogenase (GAPDH; 1:500, 60004-1-Ig, Proteintech Group, Chicago, IL). Horseradish peroxidase-conjugated AffiniPure goat anti-mouse IgG (1:5000, SA00001-1; Proteintech Group) or goat anti-rabbit IgG (1:5000, SA00001-2; Proteintech Group) were used as secondary antibodies.

### Statistical analysis

For statistical analysis of the 16S rRNA gene sequencing data, normal, and non-normally distribution data were tested using the PROC MIXED and PROC GLIMMIX procedure implemented using SAS software (SAS Institute Inc., Cary, NC). LEfSe was used to identify indicator bacteria that differentiated the treatment-specific microbiota features, and LDA was performed to estimate the effect size of each feature. A significance level (alpha) of 0.05 and an effect size threshold of 3 was used for all indicators discussed in this study. Other data from the *in vivo* and *in vitro* experiments were analyzed using the PROC MIXED or analysis of variance (ANOVA) procedures. Differences between the least-square means were compared using Tukey's honestly significant difference *post-hoc* test and were considered significant at a significance level of 0.05. The data were visualized using GraphPad Prism 5 software (Graphpad Software Inc., San Diego, CA).

### Accession number

The raw high-throughput sequencing data have been deposited in the US National Center for Biotechnology Information (NCBI) Sequence Read Archive database under the accession number SRR5860769.

## Results

### High-throughput sequencing and quality control

**Figure 1:**
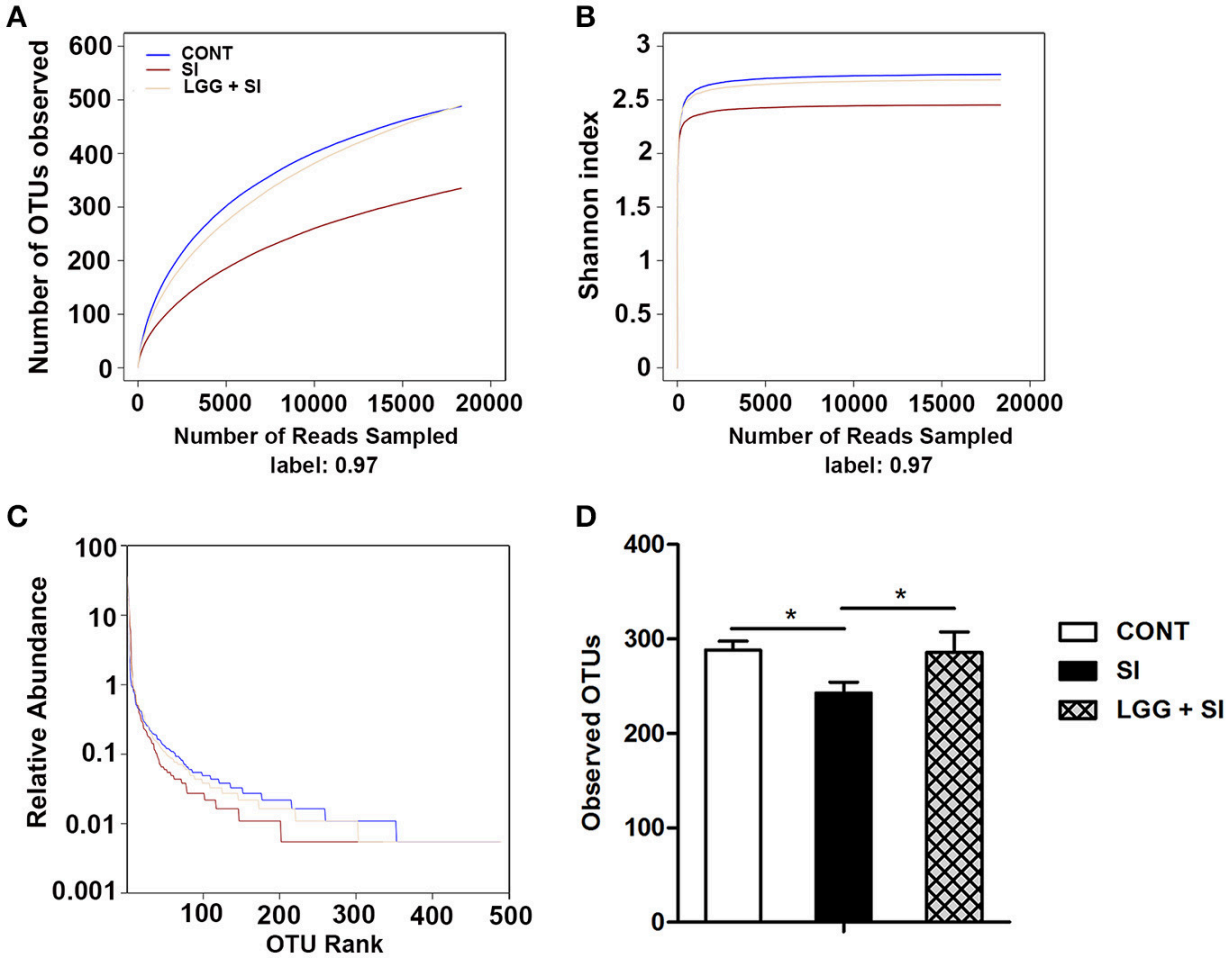
Diversity analysis of ileal mucosal microbiota. The rarefaction abundance curves **(A)**, Shannon diversity curves **(B)**, and rank abundance curves **(C)** were used to estimate the ileal mucosal microbiota diversity at 97% similarity in pigs (*n* = 7 per group) that received oral sterile physiological saline (CONT), oral sterile physiological saline followed by *S*. Infantis (5 × 10^10^ CFU/ml, 10 ml) challenge (SI), or LGG (1 × 10^9^ CFU/ml, 10 ml/day) for 1 week followed by *S*. Infantis challenge (LGG+SI). **(D)** The difference in operational taxonomic units (OTUs) observed among the three groups was analyzed using one-way ANOVA (mean ± SEM, *n* = 7 per group). ^*^P < 0.05.

After quality trimming and chimera checking, a total of 564,559 high-quality 16S rRNA gene reads were obtained from the 21 ileal mucosal samples, with a median sequence read length of 448 base pairs and a mean ± *SD* of 26,884 ± 5,513 reads per sample. This sequencing depth almost reflected the total microbial species richness, as shown by the group-based rarefaction abundance and Shannon diversity curves (Figures 1A,B). Across all samples, a total of 778 bacterial operational taxonomic units (OTUs) were identified, with a mean ± *SD* of 272 ± 44 OTUs per sample. As shown in rank abundance curves (Figure 1C), a small number of OTUs dominated and the majority of OTUs were present at low abundance in the ileal mucosal microbiota. The group-based Shannon diversity, rarefaction, and rank abundance curves showed that the CONT and LGG+SI groups had greater taxon richness than the SI group (Figures 1A–C). One-way ANOVA also showed a lower number of observed OTUs in the SI group than the CONT group (*P* = 0.037) and LGG+SI group (*P* = 0.041, Figure 1D).

### Distribution of different taxa in the ileal mucosal microbiota samples

**Figure 2:**
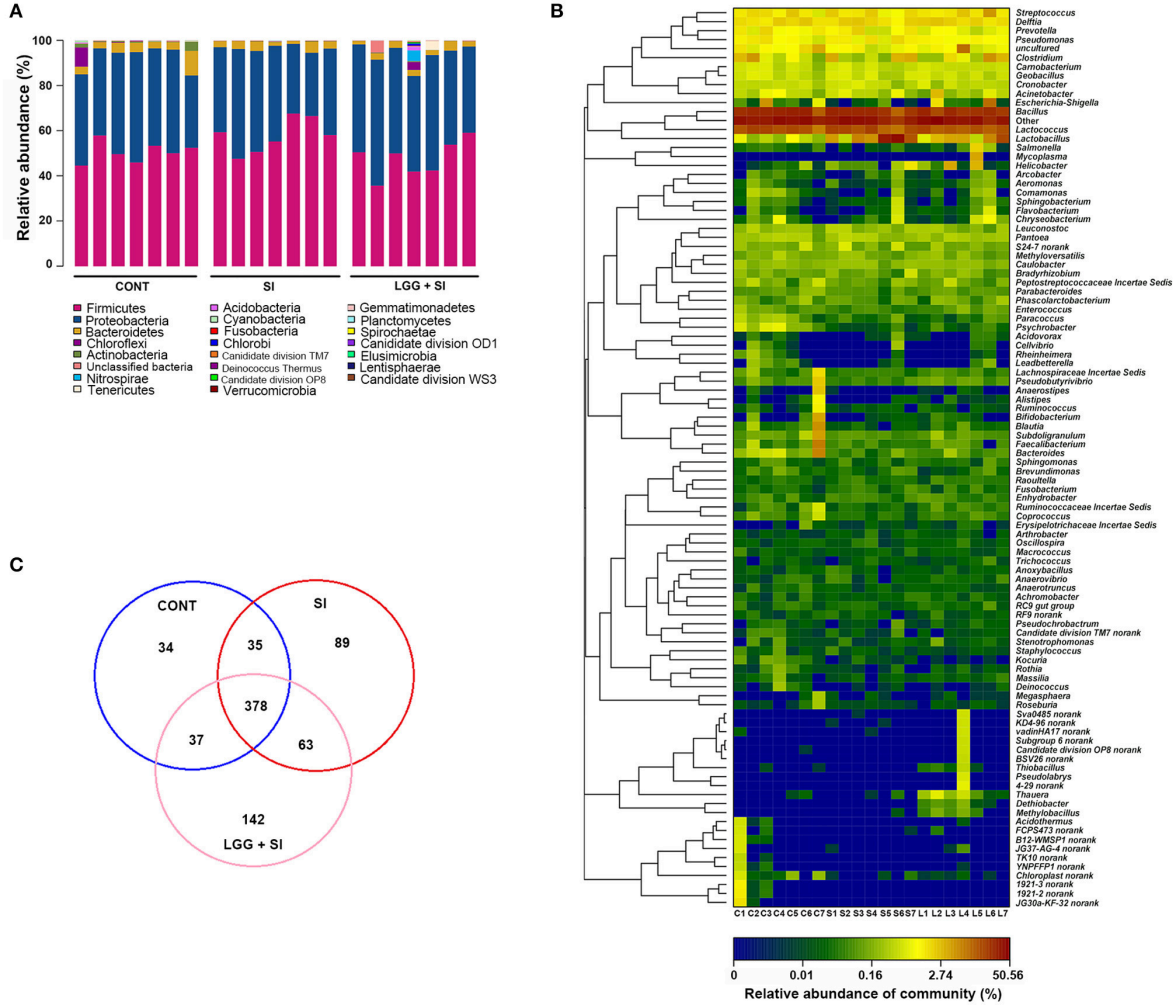
Ileal mucosal microbiota profiles of *S*. Infantis-infected pigs pretreated with LGG. **(A)** Ileal mucosal microbiota profiles at the phylum level. The stacked bars show the combined relative abundance of phylum-level OTUs per pig. Colors are assigned for all phyla detected. **(B)** Heatmap showing the spatial distributions of genera, indicating the relative abundance of the top 100 genera per pig in the CONT (C), SI (S), and LGG+SI (L) groups. Genera are clustered to the left based on relative abundance. The relative abundance of each genus is indicated by a color gradient from blue (low abundance) to red (high abundance). The letters (C, S, or L) combined with single digits (1 to 7) represent the individual pigs in the corresponding groups. **(C)** Venn diagrams illustrating overlapping OTUs in the ileal mucosal microbiota of the three groups (*n* = 7 per group).

The taxon-based analysis identified 206 families within 22 phyla (including four candidate divisions, TM7, OP8, OD1, and WS3). Regardless of the treatment group, the ileal mucosal microbiota samples were dominated by *Firmicutes* and *Proteobacteria* populations, which accounted for 52 and 42% of all the sequences, respectively (Figure 2A). In addition, *Bacteroidetes* and *Actinobacteria* were present in all the ileal mucosal samples and represented 3.38 and 0.45% of all the sequences, respectively. Eighteen other rare phyla were identified, with 13, 12, and 15 phyla in the CONT, SI, and LGG+SI groups, respectively.

In total, 337 genera were identified across all the samples. An unclassified genus within the family *Comamonadaceae* accounted for 34% of all the sequences. The 9 other most abundant genera (with a mean relative abundance >1%) accounted for 52% of all the sequences (Figure 2B), comprising *Bacillus* (23.08%), *Lactococcus* (13.95%) and *Streptococcus* (1.80%), *Lactobacillus* (6.78%), *Delftia* (2%), *Clostridium* (1.66%), *Pseudomonas* (1.22%), and *Prevotella* (1.10%). The heat map-based analysis showed there was an obvious shift in the ileal mucosal microbiota in the LGG+SI group, with an increase in the genera *Thauera, Dethiobacter, Methylobacillus*, and *Thiobacillus*, compared to the CONT and SI groups.

A high proportion of reads could not be classified at the species level. Therefore, a taxon-independent analysis using OTUs was performed. Compared with a total of 127, 107, and 119 classified species (total 143 across all samples), a total of 565, 484, and 620 OTUs were identified in the CONT, SI, and LGG+SI groups, respectively (Figure 2C). A total of 378 OTUs were shared by all the pigs, accounting for 48.59% of the total OTUs and 66.18% of all the sequences. These 378 OTUs were classified into the phyla *Firmicutes, Proteobacteria*, and *Bacteroidetes*, accounting for 35.22, 28.56, and 1.97% of all the sequences, respectively. Unique OTUs accounted for a mean of 4.37 and 18.25% of the total OTUs in the SI and LGG+SI groups, respectively, involving just 192 and 3,841 sequences, respectively.

### LGG pretreatment affected the community structure of the ileal mucosal microbiota during *S*. infantis infection

**Figure 3:**
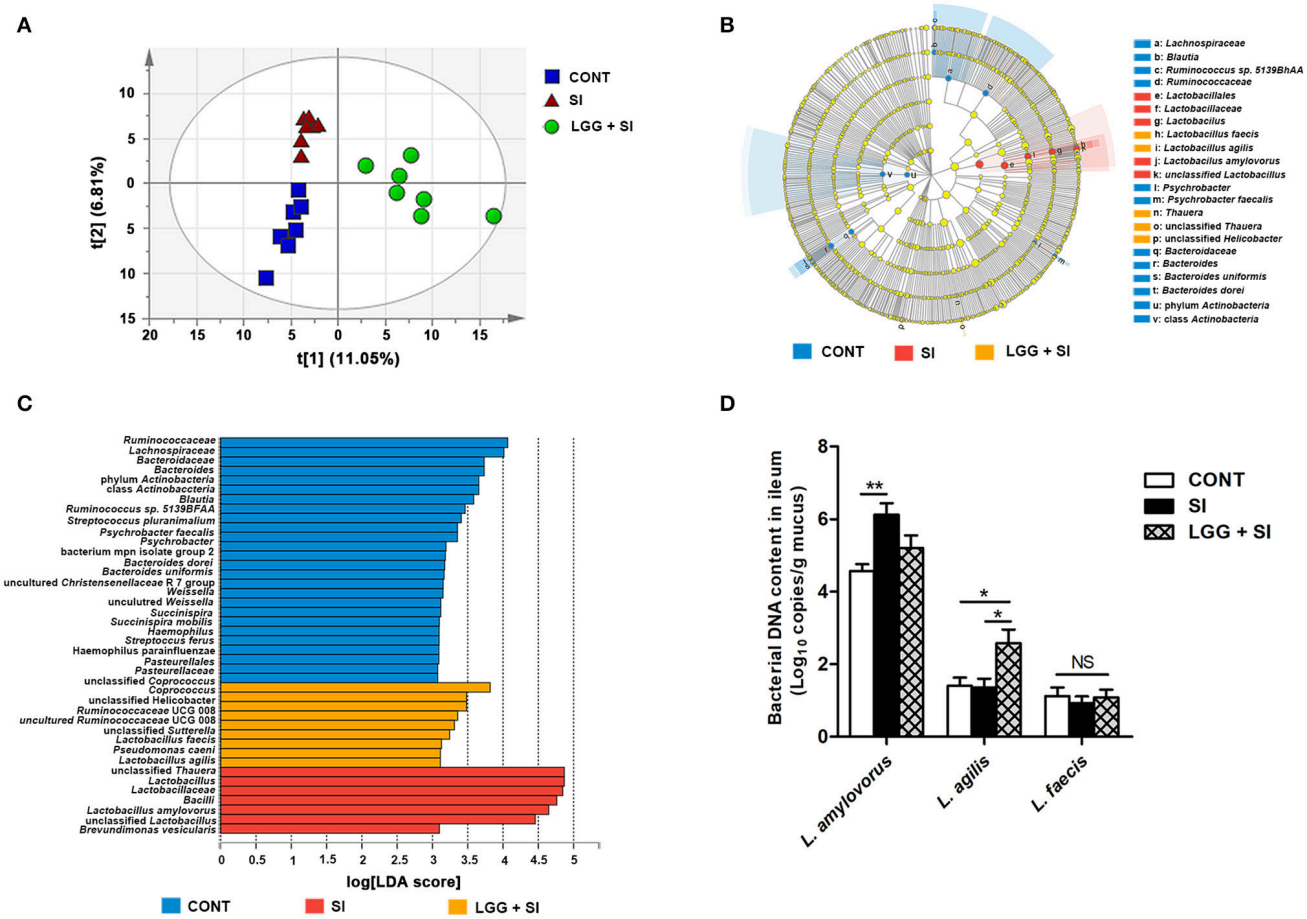
LGG pretreatment altered the ileal mucosal microbiota structure during *S*. Infantis challenge. **(A)** Two-dimensional partial least squares-discriminant analysis (PLS-DA) score plots based on the relative abundance of OTUs, showing the differences in the ileal mucosal microbiota in response to LGG pretreatment and *S*. Infantis challenge. LEfSe was performed to identify the most differentially abundant taxa in each of the three groups (*n* = 7 per group). **(B)** Taxonomic cladogram indicating the phylogenetic distribution of microbial lineages associated with the three groups (*n* = 7 per group). Lineages with an LDA value >3 are displayed. The diameter of each dot is proportional to its effect size. **(C)** Only taxa with an LDA value >3 are shown. **(D)** Quantitative PCR of bacterial 16S rRNA gene in ileum, using primers specific for *Lactobacillus* species. Results are presented as log_10_ copies/g mucus (mean ± SEM, *n* = 7 per group). ^*^P < 0.05, ^**^P < 0.01.

The ileal mucosal microbiota was altered by the treatments, as shown by an analysis of similarity (*P* = 0.001, *R* = 0.268) and a permutational multivariate analysis of variance (*P* = 0.048, *R*^2^ = 0.148) based on unweighted UniFrac distances from all the OTUs. Moreover, the PLS-DA based on the relative abundances of OTUs also showed that the three groups were well-separated, indicating clearly distinguishable ileal mucosal microbiota samples among the three groups [R^2^X(cum) = 0.462, R^2^Y(cum) = 0.995, Q^2^(cum) = 0.683; Figure 3A].

The LEfSe results showed that in the CONT pigs, four lineages had an LDA value of 3 or higher, namely, *Lachnospiraceae* (one species within the genus *Ruminococcus*), *Ruminococcaceae*, class *Actinobacteria* and *Bacteroidetes* (genera *Bacteroides uniformis* and *Bacteroides dorei*) (Figure 3B). Compared to the CONT group, in the SI group, the genera *Lactobacillus* (mainly *Lactobacillus amylovorus*) was enriched and had an LDA value >4, while *Lactobacillus agilis* and *Lactobacillus faecalis* were increased in the LGG+SI group and had an LDA value >3 (Figure 3C). Further, quantitative PCR results showed that compared with that in the CONT pigs, the total abundance of ileal *Lactobacillus amylovorus* increased (*P* = 0.005) in the SI pigs (Figure 3D). Compared to the CONT and SI pigs (*P* = 0.029, and *P* = 0.023), the LGG+SI pigs had higher abundance of *L. agilis*. No changes in the abundance of *L. faecalis* were observed among the three groups. One-way ANOVA also showed that *S*. Infantis challenge led to a 30.4-fold increase in the relative abundance of the genus *Lactobacillus* compared to the CONT group, while LGG pretreatment attenuated this increase (with an increase of 10.3-fold compared to the CONT group) (Table 1). Compared to the CONT and SI pigs, the LGG+SI pigs had higher relative abundances of the classes *Clostridia* (*Anaerobranca* and *Dethiobacter*)*, Betaproteobacteria* (*Azoarcus, Thauera*, and *Methylobacillus flagellates*) and *Gammaproteobacteira* (*Alishewanella* and *Thioalkalivibrio sulfidophilus*), and the genus *Fontibacter* within the class *Cytophagia* (Table 1).

**Table 1:** Ileum mucosal microbiota taxa alterations in response to *S*. Infantis challenge and LGG pretreatment (relative abundance, %).

**Taxa^a^**	**Treatment**^b^		
	**CONT**	**SI**	**LGG+SI**
**PHYLUM**			
*Firmicutes*	50.61^a^	57.91^b^	47.65^a^
**CLASS**			
*Bacilli*	42.44^a^	54.88^b^	44.14^a^
**ORDER**			
*Alteromonadales*	0.0023^a^	0.0008^a^	0.039^b^
*Lactobacillales*	17.38^a^	31.53^b^	21.4^a^
*Methylophilales*	0.0055^a^	0.0031^a^	0.0717^b^
*Micrococcales*	0.1995^b^	0.0982^a^^b^	0.0694^a^
*Propionibacteriales*	0.0086^b^	0.0008^a^	0.0008^a^
*Rhodocyclales*	0.1987^a^	0.1901^a^	0.512^b^
**FAMILY**			
*Alteromonadaceae*	0^a^	0^a^	0.0343^b^
*Clostridiales XIV Incertae Sedis*	0^a^	0^a^	0.0265^b^
*Cyclobacteriaceae*	0^a^	0^a^	0.007^b^
*Ectothiorhodospiraceae*	0^a^	0^a^	0.0327^b^
*Hyphomicrobiaceae*	0.0109^b^	0.0008^a^	0.0047^a^^b^
*Intrasporangiaceae*	0.0203^b^	0.0008^a^	0.0039^a^
*Lactobacillaceae*	0.4886^a^	14.84^c^	5.01^b^
*Methylophilaceae*	0.0055^a^	0.0031^a^	0.0717^b^
*Micrococcaceae*	0.1582^b^	0.0616^a^^b^	0.0499^a^
*Nocardioidaceae*	0.0086^b^	0.0008^a^	0.0008^a^
*Rhodocyclaceae*	0.1987^a^	0.1901^a^	0.5120^b^
*Syntrophomonadaceae*	0^a^	0^a^	0.0639^b^
**GENUS**			
*Alishewanella*	0^a^	0^a^	0.0343^b^
*Anaerobranca*	0^a^	0^a^	0.0265^b^
*Arsenicicoccus*	0.0094^b^	0.0008^a^	0^a^
*Azoarcus*	0^a^	0^a^	0.0281^b^
*Dethiobacter*	0^a^	0^a^	0.0639^b^
*Devosia*	0.0109^b^	0.0008^a^	0.0047^a^^b^
*Fontibacter*	0^a^	0^a^	0.007^b^
*Janibacter*	0.0109^b^	0^a^	0.0039^a^^b^
*Kocuria*	0.0764^b^	0.0132^a^	0.0023^a^
*Lactobacillus*	0.4886^a^	14.84^c^	5.01^b^
*Methylobacillus*	0^a^	0.0023^a^	0.0717^b^
*Nocardioides*	0.0086^b^	0.0008^a^	0.0008^a^
*Paracoccus*	0.2213^b^	0.0584^a^	0.0522^a^
*Psychrobacter*	0.4184^b^	0.0218^a^	0.0429^a^
*Thauera*	0.0055^a^	0.0070^a^	0.2634^b^
*Thioalkalivibrio*	0^a^	0^a^	0.0327^b^
*Weissella*	0.01251^b^	0.0023^a^	0^a^
*Rhodobacteraceae* unclassified (OTU385)	0^a^	0.0031^a^	0.1192^b^
**SPECIES**			
*Arsenicicoccus bolidensis*	0.0094^b^	0.0008^a^	0^a^
*Flavonifractor plautii*	0^a^	0^a^	0.007^b^
*Kocuria carniphila*	0.0764^b^	0.0133^a^	0.0023^a^
*Lactobacillus reuteri*	0.0935^a^	0.8151^b^	0.4231^a^^b^
*Methylobacillus flagellates*	0^a^	0.0023^a^	0.0717^b^
*Psychrobacter faecalis*	0.413^b^	0.0156^a^	0.0382^a^
*Sphingomonas aurantiaca*	0.0140^b^	0^a^	0.0031^a^^b^
*Streptococcus pluranimalium*	0.0086^b^	0^a^	0.0008^a^
*Thioalkalivibrio sulfidophilus*	0^a^	0^a^	0.0327^b^
Uncultured *Alishewanella* (OTU72)	0^a^	0^a^	0.0343^b^
Uncultured *Anaerobranca* (OTU113)	0^a^	0^a^	0.0265^b^
Unclassified *Paracoccus* (OTU263)	0.2127^b^	0.0569^a^	0.0522^a^
Unclassified *Ruminococcus* (OTU271)	0.0039^b^	0^a^	0.0008^a^^b^
Unclassified *Devosia* (OTU333)	0.0109^b^	0.0008^a^	0.0047^a^^b^
Uncultured *Thauera* (OTU407)	0.0055^a^	0.007^a^	0.2634^b^
Unclassified *Dethiobacter* (OTU515)	0^a^	0^a^	0.0639^b^
Uncultured *Weissella* (OTU567)	0.0125^b^	0.0023^a^	0^a^
Uncultured *Azoarcus* (OTU637)	0^a^	0^a^	0.0281^b^
Unclassified *Succinispira* (OTU713)	0.0055^b^	0^a^	0^a^
Uncultured *Janibacter* (OTU763)	0.0109^b^	0^a^	0.0039^a^^b^
Uncultured *Nocardioides* (OTU766)	0.0086^b^	0.0008^a^	0.0008^a^

a Taxa at the phylum, class, order, family, genus, and species levels.

b *Pigs were assigned to three groups (n = 7 per group) and each group received a different treatment, as follows: (1) CONT group, oral administration of sterile physiological saline; (2) SI group, oral administration of sterile physiological saline and oral challenge with mCherry-S. Infantis (5.0 × 10^10^ CFU/ml, 10 ml); (3) LGG+SI group, oral administration of L. rhamnosus (1 × 10^9^ CFU/ml, 10 ml/day) and oral challenge with mCherry-S. Infantis (5.0 × 10^10^ CFU/ml, 10 ml). At 0900 a.m., on days 1 to 7, the pigs in the LGG+SI group were intragastrically treated with 10 ml LGG solution daily, while pigs in the CONT and SI groups were pretreated with an equal volume of sterile physiological saline. At 0900 a.m. on day 8, the pigs in groups SI and LGG+SI were orally challenged with 10 ml mCherry-S. Infantis, whereas pigs in the CONT group received 10 ml sterile physiological saline*.

### LGG pretreatment decreased intracellular invasion and IPEC-J2 cell death caused by *S*. infantis and inhibited *S*. infantis translocation to the liver

An IPEC-J2 cell model of LGG and *S*. Infantis co-incubation was established. *S*. Infantis was susceptible to NAL but LGG was resistant to NAL (Figure S1A). Furthermore, 10 μg/ml of NAL inhibited the growth of *S*. Infantis (3.56 × 10^6^ CFU/ml), and *S*. Infantis growth on the recovery medium ceased when the concentration of the NAL was 50 μg/ml (Figures S1B,C). NAL at a range of 0–200 μg/ml did not affect the growth of LGG (Figure S1D). Thus, in the IPEC-J2 cells, 50 μg/ml of NAL was used to kill the extracellular non-internalized *S*. Infantis and 10 μg/ml of NAL was used to restrict the growth of extracellular *S*. Infantis that escaped from the damaged IPEC-J2 cells for the remainder of the infection.

**Figure 4:**
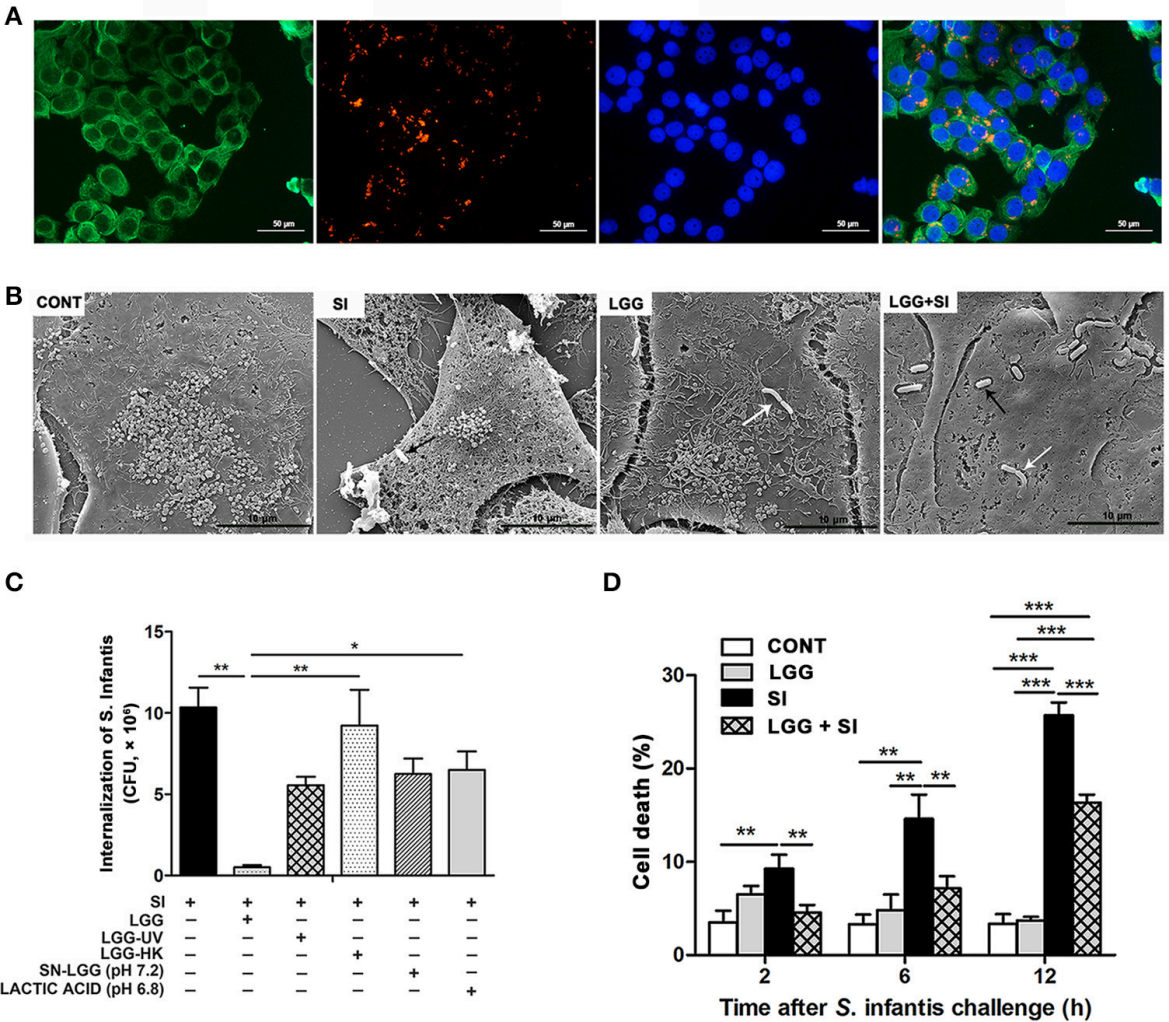
LGG pretreatment decreased intracellular invasion and IPEC-J2 cell death caused by *S*. Infantis and inhibited *S*. Infantis translocation to the liver. IPEC-J2 cells were treated with medium alone (CONT), *L. rhamnosus* GG alone (LGG), *S*. Infantis or mCherry-*S*. Infantis (SI), or they were preincubated with *L. rhamnosus* GG for 2 h followed by *S*. Infantis challenge (LGG+SI). **(A)** Immunofluorescence analysis of the *S*. Infantis internalization (using mCherry-*S*. Infantis) in the IPEC-J2 cells. IPEC-J2 cells were stained with mouse anti-cytokeratin-18 (green) and DAPI (blue). Scale bar, 50 μm. **(B)** IPEC-J2 cell ultrastructure observed using scanning electron microscopy. Black arrows indicate *S*. Infantis and white arrows indicate LGG. Scale bar, 10 μm. **(C)** IPEC-J2 cells from the indicated cultures. An internalization assay using *S*. Infantis alone serves as a reference. The number of *S*. Infantis recovered from the IPEC-J2 cells was determined. **(D)** The IPEC-J2 cell death was determined using 0.4% Trypan blue dye. The cell death rate is presented as the ratio of the number of dead cells to the total number of cells. The data are presented as mean ± SEM of three independent experiments. ^*^P < 0.05, ^**^P < 0.01, ^***^P < 0.001.

Intracellular *S*. Infantis invasion was observed in the IPEC-J2 cells (Figure 4A). Under a scanning electron microscope, the untreated control IPEC-J2 cells appeared smooth, with intact cell membranes, and numerous microvilli. At 6 h after *S*. Infantis challenge, *S*. Infantis could be seen attached to the surface of the IPEC-J2 cells. The IPEC-J2 cells exhibited apparent damage: the cell membranes were disrupted and microvilli became less evident. The cells exhibited atrophy and various degrees of collapse (Figure 4B). Pretreatment with LGG reduced the degree of cell membrane disruption for up to 6 h after *S*. Infantis infection.

At 2 h after *S*. Infantis challenge, the number of internalized *S*. Infantis was about 10.3 × 10^6^ CFU. Pretreatment with live LGG led to a decrease in the rate *S*. Infantis internalization to 4.8% (*P* = 0.001), but this inhibitory effect was not observed in cell cultures pretreated with ultraviolet-irradiated LGG, heat-killed LGG, LGG supernatant, or DMEM acidified with lactic acid (Figure 4C). *S*. Infantis infection resulted in an increase in the percentage of dead cells at 2, 6, and 12 h, but this increase was attenuated by LGG (Figure 4D).

### LGG pretreatment attenuated *S*. infantis-induced autophagy and increased EGFR and Akt activation in the ileum of pigs

**Figure 5:**
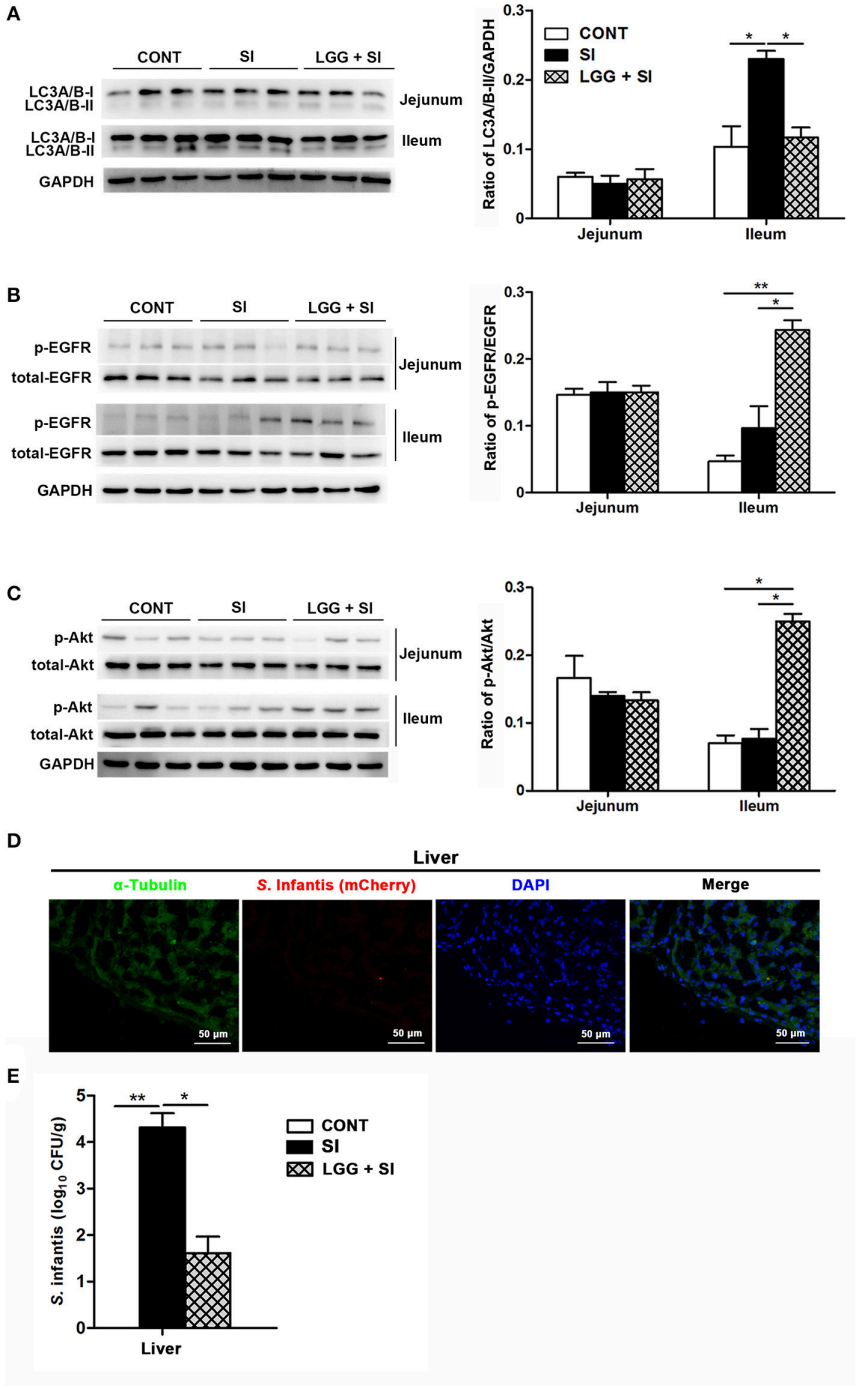
LGG pretreatment attenuated *S*. Infantis-induced autophagy and increased EGFR and Akt activation in the ileum of pigs. Representative panels of LC3A/B-I and LC3A/B-II **(A)**, p-EGFR and total-EGFR **(B)**, and p-Akt and total-Akt **(C)** proteins in the jejunum and ileum tissues collected from pigs at 10 days after *S*. Infantis challenge (left panel). Each band represents an individual pig. The results are presented as the ratios of the band intensities of LC3A/B-II to GAPDH, p-Akt to total-Akt and p-EGFR to total-EGFR (right panel). **(D)** Immunofluorescence analysis of the recovered *S*. Infantis (mCherry) in the liver. Frozen sections of liver from pigs challenged with *S*. Infantis were stained with anti-pig α-tubulin (green) and DAPI (blue). Scare bars, 50 μm. **(E)** The number of live *S*. Infantis recovered from the livers of the pigs was determined. The data are presented as mean ± SEM (*n* = 7 per group). ^*^P < 0.05, ^**^P < 0.01.

*S*. Infantis challenge resulted in an increase in LC3A/B-II protein expression in the ileum (*P* = 0.032), but this increase was attenuated by LGG pretreatment (*P* = 0.041, Figure 5A). Compared with the CONT and SI pigs, phosphorylated EGFR (pEGFR; *P* = 0.008 and *P* = 0.022, respectively) and phosphorylated Akt (p-Akt; *P* = 0.010 and *P* = 0.018, respectively) protein expression were elevated in the ileum of the LGG+SI pigs (Figures 5B,C). No differences were found in the protein expression of LC3A/B-II, p-EGFR and p-Akt in the jejunum of pigs in any of the three groups.

After *S*. Infantis challenge, *S*. Infantis could translocate from the intestine into the liver (Figure 5D). Decreased recovery of viable *S*. Infantis in the liver was observed in pigs pretreated with LGG compared with SI pigs (*P* = 0.021, Figure 5E). *S*. Infantis was not recovered in the spleens of pigs in any of the three groups.

### LGG pretreatment attenuated *S*. infantis-induced autophagy and promoted EGFR and Akt activation in the IPEC-J2 cell monolayers

**Figure 6:**
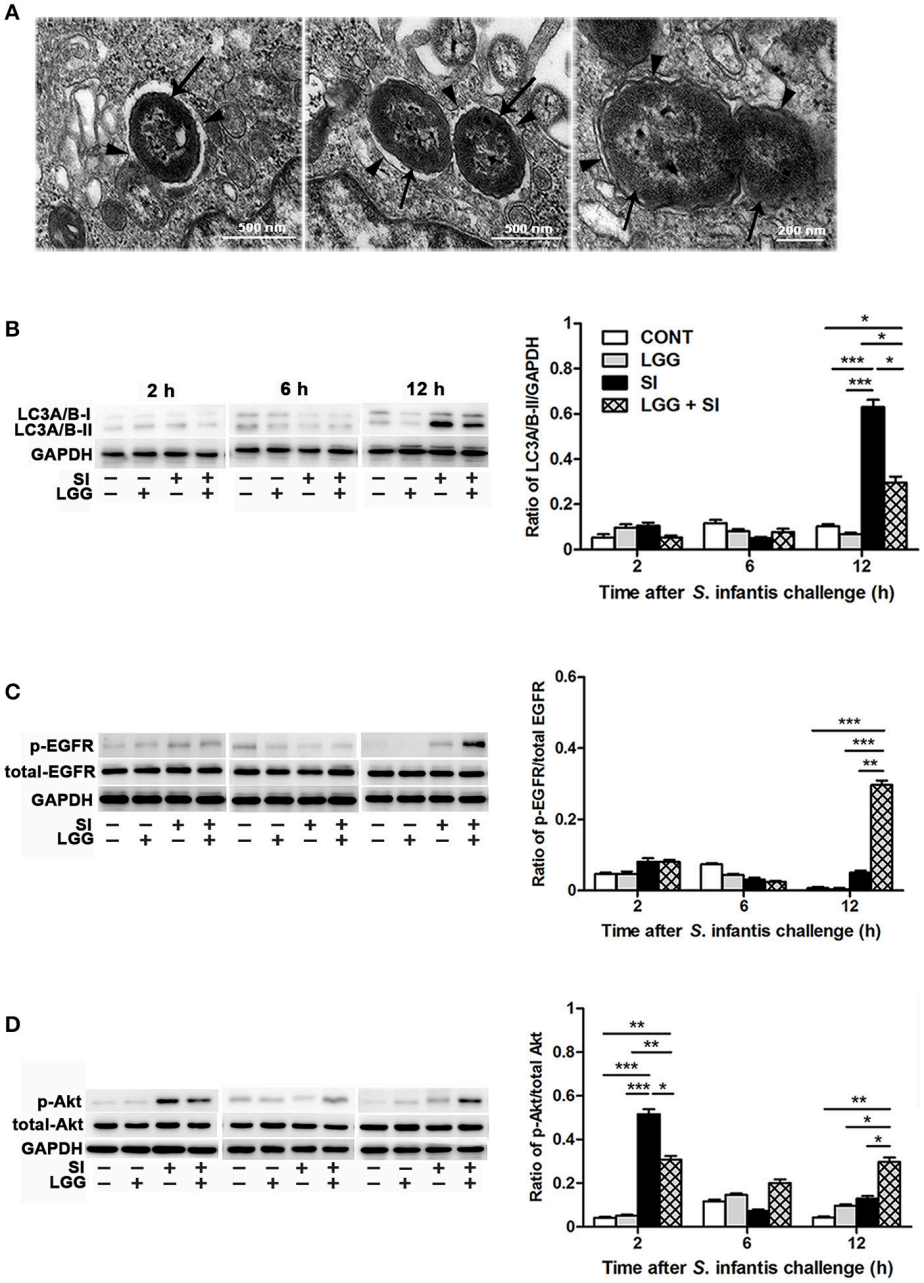
LGG pretreatment attenuated *S*. Infantis-induced autophagy and promoted EGFR and Akt activation in the IPEC-J2 cell monolayers. **(A**) Autophagy in IPEC-J2 cells infected with *S*. Infantis was observed using transmission electron microscopy. Black arrows indicate *S*. Infantis and black arrowheads indicate single or double membrane of autophagosomes. Representative panels of LC3A/B-I and LC3A/B-II **(B)**, p-EGFR and total-EGFR **(C)**, and p-Akt and total-Akt **(D)** proteins in IPEC-J2 cells collected at the indicated time points after *S*. Infantis challenge (left panel). Each band represents an individual pig. The results are presented as the ratios of the band intensities of LC3A/B-II to GAPDH, p-Akt to total-Akt and p-EGFR to total-EGFR (right panel). The data are presented as mean ± SEM of three independent experiments. ^*^P < 0.05, ^**^P < 0.01, ^***^P < 0.001.

NAL and its solvent dimethylsulfoxide did not lead to autophagy of the IPEC-J2 cells (Figure S1E). Under a conventional transmission electron microscope, *S*. Infantis was found to be contained within single or double membranes, which were characteristic of autophagosomes (Figure 6A). Further, the Western blotting analysis showed that, at 12 h, the IPEC-J2 cells only exposed to *S*. Infantis had higher LC3A/B-II protein expression than the untreated control cells and cells only pretreated with LGG, while LGG pretreatment followed by *S*. Infantis infection resulted in a decrease in LC3A/B-II protein expression in comparison to the cells only exposed to *S*. Infantis (Figure 6B). At 12 h, LC3A/B-II protein expression was higher in cells pretreated with LGG followed by *S*. Infantis infection than in the untreated control cells and cells only pretreated with LGG alone. At 2 and 6 h (after *S*. Infantis infection or the equivalent time point for the other cells), LC3A/B-II protein expression was unchanged among the four IPEC-J2 cell groups.

Expression of p-EGFR protein was elevated at 12 h after *S*. Infantis infection in cells pretreated with LGG compared with the untreated control cells (*P* < 0.001), cells infected with *S*. Infantis and cells pretreated with LGG alone (*P* < 0.001, Figure 6C). At 2 h after *S*. Infantis infection, compared with the untreated control cells and cells pretreated with LGG alone, expression of p-Akt protein was increased in cells infected with *S*. Infantis (*P* < 0.001, Figure 6D), but this increase was inhibited by pretreatment with LGG (*P* = 0.014). There was a higher expression of p-Akt protein in cells pretreated with LGG compared with untreated control cells at 2 and 12 h after *S*. Infantis infection (*P* = 0.002 and *P* = 0.008, respectively).

## Discussion

Pork products contaminated with *S*. Infantis are a globally important source of foodborne gastroenteritis in humans (Schroeder et al., 2016). In a previous study, we found that oral inoculation of newly weaned piglets with *S*. Infantis results in fever, diarrhea, and small intestine enteritis, but administration of the probiotic LGG reduces the severity of diarrhea and alleviates intestinal inflammation caused by *S*. Infantis (Yang et al., 2017). The present study showed that LGG prevented *S*. Infantis-induced loss of microbial diversity and restored the gut mucosal microbiota balance. Furthermore, administration of LGG decreased *S*. Infantis internalization and inhibited *S*. Infantis-induced IEC autophagy by promoting EGFR-mediated activation of the negative mediator Akt, which in turn suppressed the death of the IECs and thus restricted systemic *S*. Infantis infection.

Dysbiosis of the gut microbiota has emerged as a leading cause of post-weaning diarrhea and associated infections in piglets (Gresse et al., 2017). In the present study, we observed an aberrant microbiota pattern, with a decrease in α diversity, in piglets challenged with *S*. Infantis. Loss of gut microbiota diversity often occurs during enteric pathogen infections and, in turn, it could increase the host susceptibility to *Salmonella*-induced colitis (Ferreira et al., 2011). In a previous study, mice harboring a gut microbiota with low complexity failed to clear *S*. Typhimurium (Endt et al., 2010). Restoration of lost gut microbiota diversity can inhibit *S*. Typhimurium infection and growth (Malo et al., 2010). In the present study, administration of LGG prevented the loss of microbiota diversity caused by *S*. Infantis, which was attributed to increases in both the relative abundance of species and the number of species. Indeed, more species were exclusively found in piglets pretreated with LGG than in the other two groups. Our results indicate that LGG may serve as a gut microbiota-related therapeutic strategy for controlling enteric pathogen infections in piglets by restoring the microbial balance associated with weaning transition.

The collateral effects of *S*. Infantis challenge included the dramatic 30.4-fold increase in *L. amylovorus* populations, whereas *L. agilis* and *L. faecalis* were enriched by 10.3-fold in the pigs pretreated with LGG. The ANOVA results also showed that administration of LGG attenuated the *S*. Infantis-induced *Lactobacillus* expansion.

*Lactobacillus* species are the one of the core genera found in the swine gut and generally recognized as safe (Valeriano et al., 2017). In piglets, dietary supplementation with chlortetracycline drives a specific shift in the ileal microbiota from an *L. johnsonii*-dominated microbiota to an *L*. *amylovorus*-dominated microbiota (Rettedal et al., 2009). *L. amylovorus* is considered as a beneficial microbe to reduce piglet diarrhea via the activities of inhibiting pathogen adhesion and immunomodulatory capacity (Finamore et al., 2014). However, Lactobacilli have species/strain-specific characteristics in response to external factors such as defense against pathogen infections. A meta-analysis revealed that the effectiveness of pathogen infection prevention strategies using probiotic supplements is associated with specific probiotic species, including *Lactobacillus* and *Bifidobacterium* species (Feng et al., 2017). *Lactobacillus* has been reported to result in infections in immunocompromised patients (Land et al., 2005). Our previous studies also showed that administration of a 100-times higher dose of *L. rhamnosus* to piglets may reduce the prophylactic effect of *L. rhamnosus* against enterotoxigenic *E. coli* K88 infection (Li et al., 2012; Zhu et al., 2014). An increased abundance of *Lactobacillus* species is positively correlated with a predisposition for developing *Salmonella*-induced colitis (Ferreira et al., 2011). The probiotic strains that pose potential risks are most likely the strains with higher distributions in the mucus zone (Liu et al., 2016). Compared to *L. amylovorus, L. agilis* exhibits lower adhesion to porcine intestinal mucin (Kinoshita et al., 2016). Although the mechanism behind the *S*. Infantis-induced rise in *Lactobacillus* populations remains unclear, our data suggest that the resultant dramatic *L. amylovorus* bloom increases the risk of opportunistic infection of the inflamed gut of piglets challenged with *S*. Infantis. Administration of exogenous LGG is a beneficial strategy for maintaining the balance of autochthonous probiotic *Lactobacillus* strain functions in terms of the balance between the promotion of gut health and gut dysbiosis due to excessive growth.

Apart from the increased *Lactobacillus* populations, the gut microbiota dysbiosis caused by *S*. Infantis infection was accompanied by decreased populations of some rare taxa, namely, the genera *Arsenicicoccus, Janibacter, Kocuria, Nocardioides, Devosia, Paracoccus, Psychrobacter*, and *Weissella*. We postulate that these specific changes may contribute to the detrimental effects of *S*. Infantis infection on gut health. For example, *Janibacter* exhibits biocontrol potential against certain fungal pathogens (Nimaichand et al., 2015). In neonatal piglets treated with sodium butyrate, expansion of *Kocuria* contributes to decreased levels of proinflammatory interleukin 6 and 8 and interferon γ (Xu et al., 2016). Decreased abundance of *Devosia* is associated with irritable bowel syndrome in humans (Ng et al., 2013). Furthermore, increased populations of the genera *Alishewanella, Azoarcus, Thauera, Thioalkalivibrio, Methylobacillus, Anaerobranca, Dethiobacter*, and *Fontibacter* were observed in pigs pretreated with LGG. These rare taxa may represent useful novel probiotic strains for controlling enteric infection.

Autophagy is an essential mechanism of IEC-intrinsic innate immunity for promoting the clearance of *Salmonella*. IEC autophagy is specifically activated by *S*. Typhimurium via the innate immune adaptor protein MyD88, and it limits bacterial dissemination to extraintestinal tissues (Benjamin et al., 2013). In the present study, *S*. Infantis infection elicited autophagy in the intestinal epithelium of pigs, while administration of LGG inhibited autophagy. Furthermore, in the *in vitro* IPEC-J2 model of *S*. Infantis and LGG co-incubation, LGG pretreatment also suppressed IPEC-J2 cell autophagy at 12 h during *S*. Infantis infection.

Autophagic recognition of *Salmonella* in IECs is only a transient response. *Salmonella* activates autophagy by amino acid starvation-induced inhibition of downstream mTOR signaling. Subsequently, at 4 h after the initial infection, the normalized cytosolic amino acid levels relieve the inhibition of mTOR signaling and prevent autophagic targeting of bacteria (Tattoli et al., 2012a,b). Moreover, autophagy facilitates *Salmonella* replication in the cell cytosol (Yu et al., 2014). *S*. Typhimurium can obtain access to host-derived peptides for intracellular growth by co-opting the host chaperone-mediated autophagy process in human intestinal epithelial LoVo cells (Singh et al., 2017). Recent evidence suggests that *S*. Typhimurium is equipped to evade autophagic defenses and that it has evolved adaptations to protect against autophagic elimination (Owen et al., 2014).

In contrast to autophagy-induced cell survival, prolonged autophagy leads to autophagic cell death due to the degradation components that are essential for cell survival (Levine and Kroemer, 2008). In the present study, *S*. Infantis infection led to IEC damage and death, while LGG pretreatment decreased IEC death. Moreover, administration of LGG reduced the initial *S*. Infantis invasion of IPEC-J2 cells and suppressed *S*. Infantis translocation to the liver in piglets. Previous research has indicated that invasion-primed *Salmonella* bacteria are released into the intestinal lumen via epithelial cell extrusion and that they initiate secondary infections in neighboring cells (Knodler et al., 2010). A study demonstrated that the endoplasmic reticulum stress pathway contributes to autophagic death of intestinal epithelial Caco-2 cells (Tang et al., 2015). A mix of 33 probiotic bacterial strains (including several *Lactobacillus* species) reduced *S*. Typhimurium translocation to internal tissues by preserving the intestinal tight junctions (Martz et al., 2015). Our data indicate that LGG can inhibit the *S*. Infantis-induced autophagy-related death of IECs and maintain the intestinal epithelial barrier by preventing the initial *S*. Infantis invasion, thereby preventing *S*. Infantis from gaining access to the systemic circulation.

EGFR signaling is involved in regulating cellular survival; the indirect recruitment of PI3K to tyrosine-phosphorylated EGFR activates Akt (Banck et al., 2013). Activation of the PI3K/Akt pathway negatively regulates autophagy by targeting the downstream mTOR complex. In the present study, LGG pretreatment promoted the activation of EGFR and Akt in the ileum of pigs and IPEC-J2 cells during *S*. Infantis infection. Epidermal growth factor (EGF) is a ligand of EGFR, and EGF supplementation has been shown to block autophagy in IEC-6 cells and in a neonatal rat model of necrotizing enterocolitis (Maynard et al., 2010). Activation of EGFR also suppresses autophagy independently of mTOR via the direct phosphorylation and inhibition of the autophagy-related protein Beclin 1 (Wei et al., 2013). It has been reported that the LGG-derived p40 protein transactivates the EGFR/Akt signaling pathway by promoting the release of the EGFR ligand heparin-binding (HB)-EGF, and p40 has been shown to maintain the epithelial barrier (Yan et al., 2013). While the present study provides indirect evidences for the EGFR-mediated activation of the negative mediator Akt by LGG in preventing *S*. Infantis-induced intestinal autophagy, future *in vitro* studies using specific Akt inhibitors are essential for ultimate confirmation of the potential of LGG in controlling pathogen infection by promoting autophagy.

Our previous study also suggests that *L. rhamnosus* ATCC7469 promotes EGFR and Akt activation in a time-dependent manner and that it can maintain IPEC-J2 cell barrier function, thus limiting extracellular *E. coli* infection (Zhang et al., 2015). Our current data indicate that LGG administration suppressed *S*. Infantis-induced aberrant IEC autophagy by promoting EGFR-mediated activation of the negative mediator, Akt, which, in turn, inhibited IEC death and thus restricted *S*. Infantis systemic infection. However, it was also reported that probiotic *Bacillus amyloliquefaciens* induces autophagy to promote the elimination of intracellular *E. coli* in macrophages via upregulating the expression of Beclin1 and autophagy-related genes (Atg)*5-Atg12-Atg16* complex, but not Akt/mTOR signaling pathway (Wu et al., 2017). Undoubtedly, the primary result of this study require further realization with other probiotic strains to determine whether the protective mechanism is specific to LGG.

In conclusion, *S*. Infantis infection induces dysbiosis of the ileal mucosal microbiota, which is characterized by loss of microbial diversity, dramatic increase in *Lactobacillus* populations (mainly *L. amylovorus*) and decreased abundance of *Arsenicicoccus, Janibacter, Kocuria, Nocardioides, Devosia, Paracoccus, Psychrobacter*, and *Weissella*. The beneficial effect of LGG is due in part to the moderate expansion of *L. amylovorus* and *L. agilis*, as well as some members of the phyla *Proteobacteria, Firmicutes*, and *Bacteroidetes*. Administration of LGG suppresses the initial invasion of *S*. Infantis and inhibits *S*. Infantis-induced IEC autophagy by promoting EGFR-mediated activation of the negative mediator Akt, which, in turn, maintains the intestinal epithelial barrier by decreasing the death of IECs and thus restricts systemic *S*. Infantis infection. Our findings suggest the necessity of accurately defining the composition of healthy and disturbed gut microbiota in order to help to optimally select specific probiotics that may prevent enteric infections. This study expands the current understanding of the potential of using probiotics against pathogen infections.

## Author contributions

Conceived and designed the experiments: WZ, Y-HZ, and J-FW. Performed the experiments: WZ, G-YY, XL, BX, XH, and J-HS. Analyzed the data and wrote the paper: WZ, Y-HZ, and J-FW.

### Conflict of interest statement

The authors declare that the research was conducted in the absence of any commercial or financial relationships that could be construed as a potential conflict of interest. The reviewer JAG and handling Editor declared their shared affiliation.

## Supplementary material

Figure S1 Nalidixic acid screening and effect of nalidixic acid on IPEC-J2 cell autophagy. **(A)** Representative nalidixic acid inhibition zone using the Kirby Bauer disc diffusion method. Black arrows indicate nalidixic acid. **(B)** The number of live *S*. Infantis recovered from LB broth containing nalidixic acid dissolved in dimethylsulfoxide was determined. The well without nalidixic acid and dimethylsulfoxide serves as the positive reference. The effects of nalidixic acid at a range of concentrations (from 0 to 200 μg/ml) on the growth of *S*. Infantis **(C)** and LGG **(D)** were determined using spectrophotometry at 600 nm. **(E)** Representative panels of LC3A/B-I and LC3A/B-II in IPEC-J2 cells collected at the indicated time points after adding nalidixic acid. The experiments were performed in triplicate.
