# Supplementary figures and images for: Lactobacillus rhamnosus GG Affects Microbiota and Suppresses Autophagy in the Intestines of Pigs Challenged with Salmonella Infantis
Source: Front Microbiol. 2018 Jan 17;8:2705. doi: 10.3389/fmicb.2017.02705 (PMC5785727; doi:10.3389/fmicb.2017.02705)

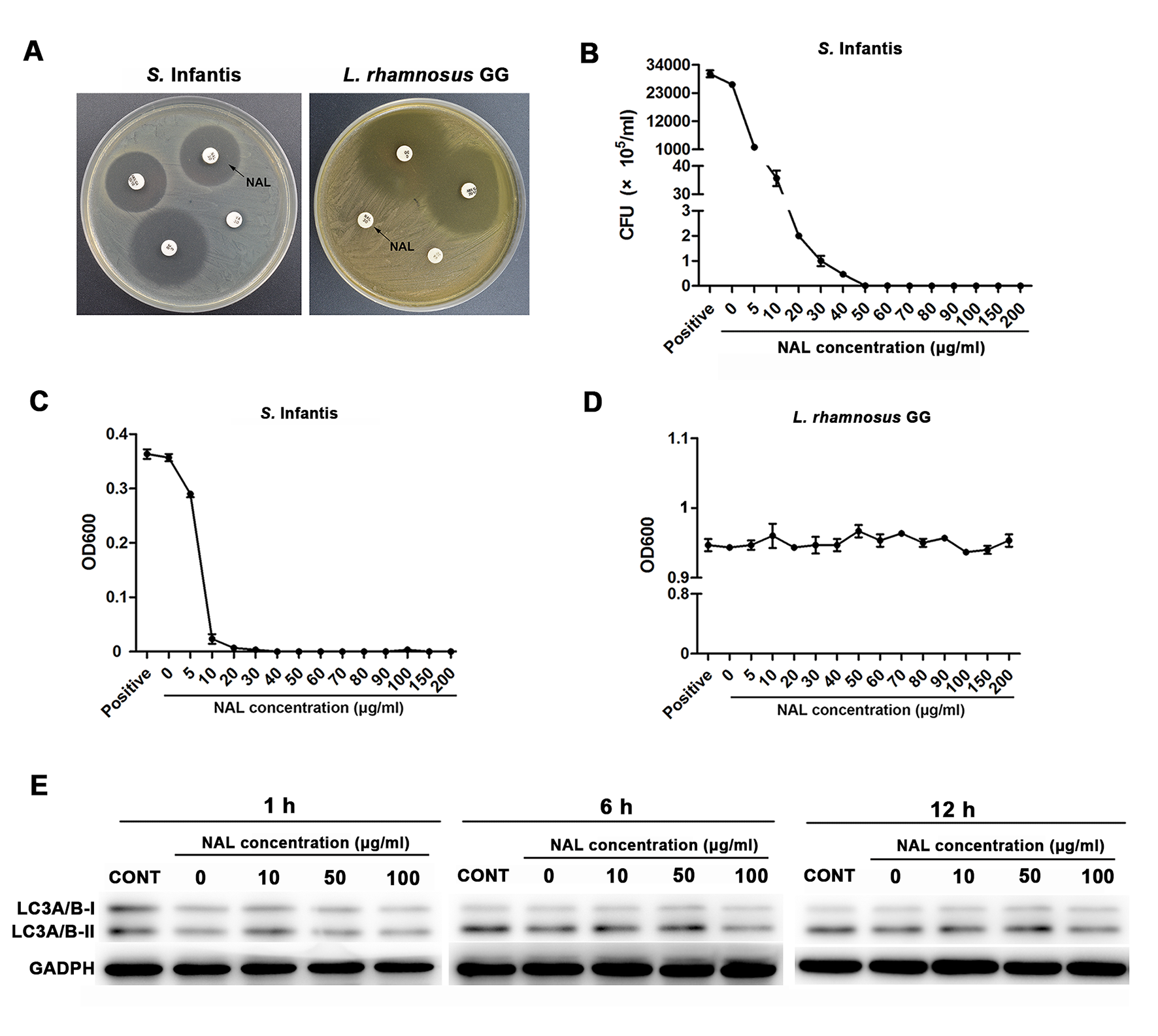

Supplement: Figure S1 — Nalidixic acid screening and effect of nalidixic acid on IPEC-J2 cell autophagy. (A) Representative nalidixic acid inhibition zone using the Kirby Bauer disc diffusion method. Black arrows indicate nalidixic acid. (B) The number of live S. Infantis recovered from LB broth containing nalidixic acid dissolved in dimethylsulfoxide was determined. The well without nalidixic acid and dimethylsulfoxide serves as the positive reference. The effects of nalidixic acid at a range of concentrations (from 0 to 200 μg/ml) on the growth of S. Infantis (C) and LGG (D) were determined using spectrophotometry at 600 nm. (E) Representative panels of LC3A/B-I and LC3A/B-II in IPEC-J2 cells collected at the indicated time points after adding nalidixic acid. The experiments were performed in triplicate. [file Image1.TIF]
